# Multi-locus variable-number tandem repeat analysis of Chinese *Brucella* strains isolated from 1953 to 2013

**DOI:** 10.1186/s40249-017-0296-0

**Published:** 2017-05-02

**Authors:** Guo-Zhong Tian, Bu-Yun Cui, Dong-Ri Piao, Hong-Yan Zhao, Lan-Yu Li, Xi Liu, Pei Xiao, Zhong-Zhi Zhao, Li-Qing Xu, Hai Jiang, Zhen-Jun Li

**Affiliations:** 1 State Key Laboratory for Infectious Disease Prevention and Control, Collaborative Innovation Center for Diagnosis and Treatment of Infectious Diseases, National Institute for Communicable Disease Control and Prevention, Chinese Center for Disease Prevention and Control, Beijing, 102206 China; 2 National Institute of Occupational Health and Poison Control, Chinese Center for Disease Prevention and Control, Beijing, 100050 China; 3 Department of Brucellosis Prevention and Control, Qinghai Institute for Endemic Disease Prevention and Control, Xining, 811602 China

**Keywords:** Brucella, MLVA, Molecular epidemiology

## Abstract

**Background:**

Brucellosis was a common human and livestock disease caused by *Brucella* strains, the category B priority pathogens by the US Center for Disease Control (CDC). Identified as a priority disease in human and livestock populations, the increasing incidence in recent years in China needs urgent control measures for this disease but the molecular background important for monitoring the epidemiology of *Brucella* strains at the national level is still lacking.

**Methods:**

A total of 600 *Brucella* isolates collected during 60 years (from 1953 to 2013) in China were genotyped by multiple locus variable-number tandem repeat analysis (MLVA) and the variation degree of MLVA11 loci was calculated by the Hunter Gaston Diversity Index (HGDI) values. The charts and map were processed by Excel 2013, and cluster analysis and epidemiological distribution was performed using BioNumerics (version 5.1).

**Results:**

The 600 representative *Brucella* isolates fell into 104 genotypes with 58 singleton genotypes by the MLVA11 assay, including *B. melitensis* biovars 2 and 3 (five main genotypes), *B. abortus* biovars 1 and 3 (two main genotypes), *B. suis* biovars 1 and 3 (three main genotypes), and *B. canis* (two main genotypes) respectively. While most *B. suis* biovar 1 and biovar 3 were respectively found in northern provinces and southern provinces, *B. melitensis* and *B. abortus* strains were dominant in China. Canine Brucellosis was only found in animals without any human cases reported. Eight Brucellosis epidemic peaks emerged during the 60 years between 1953 and 2013: 1955 – 1959, 1962 – 1969, 1971 – 1975, 1977 – 1983, 1985 – 1989, 1992 – 1997, 2000 – 2008 and 2010 – 2013 in China.

**Conclusions:**

Brucellosis has its unique molecular epidemiological patterns with specific spatial and temporal distribution according to MLVA.

**Trial registration:**

IDOP-D-16-00101.

**Electronic supplementary material:**

The online version of this article (doi:10.1186/s40249-017-0296-0) contains supplementary material, which is available to authorized users.

## Multilingual abstracts

Please see Additional file 1 for translation of the abstract into the five official working languages of the United Nations.

## Background

Brucellosis is one of the world’s most widespread zoonotic diseases. It is also seen as a reemerging infectious disease because of increasing incidence in recent years. It also is identified as a priority disease needing urgent control measures in human and livestock populations in China [1–7]. A total of 43 486 confirmed human cases were reported in 2013 (annual incidence of 3.3 cases/100 000 population) in China, and 57 222 cases were reported in 2014. Brucellosis has been found in all China.

Previous studies have confirmed that the multiple locus variable-number tandem repeat analysis (MLVA) is a useful tool for identifying and genotyping of strains and for epidemiological trace-back investigations [8]. It was also applied in *Brucella* [5–7]. The objectives of this study was to analyze the genotyping of 600 *Brucella* isolates, which were isolated from 1953 to 2013 in China by multiple locus variable-number tandem repeat analysis (MLVA) in order to more comprehensively understand the patterns of molecular epidemiology of Brucellosis in China, to trace the flow direction and variation of *Brucella*, and to provide reference information for the prevention and control of Brucellosis in China.

## Methods

### *Brucella* isolates data

There were 2 620 human and animal field *Brucella* isolates, which were collected from 29 provinces in China from 1953 to 2013. These strains came from the provincial CDC which were isolated and cultured from blood and tissues (such as liver and spleen). These isolates were identified as *Brucella* based on distinct host specificity, pathogenicity, and minor phenotypic differences based on serotyping, phage typing, fuchsin and thionin dye sensitivity, CO_2_ requirement, H_2_S production and metabolic properties [9]. They were dried and preserved in the −80 °C refrigerator in our laboratory. The stratified sampling method was used to select the number of *Brucella* isolates for this study. The definition of strata was *Brucella* bioytping. The selected proportion was 20.0% in each strata. 22.9% (600/2 620) of *Brucella* isolates were selected for this study.

### Molecular typing methods

Total genomic DNA was individually extracted from the 600 representative strains and MLVA was performed as described previously [10, 11]. The MLVA11 assay involved 11 loci including Bruce06, Bruce08, Bruce11, Bruce12, Bruce18, Bruce19, Bruce21, Bruce42, Bruce43, Bruce45, and Bruce55. A total of 11 PCR primers were labeled with 5′-fluorescent 6-FAM and PCR products were resolved by capillary electrophoresis on an ABI Prism 3130 automated fluorescent capillary DNA sequencer (Applied Biosystems). The PCR fragment sizes were converted to repeat units by following the published allele numbering system (http://mlva.u-psud.fr, *Brucella* support website for MLVA typing). All data were analyzed by using BioNumerics (version 5.1, Applied Maths, Belgium). Cluster analysis was based on the categorical coefficient and unweighted pair group method using arithmetic averages (UPGMA) method. The variation degree of MLVA11 loci was calculated by Hunter Gaston Diversity Index (HGDI) values. The charts and map were produced by Excel 2013.

## Results

### Genetic diversity of MLVA-11 loci and cluster analysis

The Hunter Gaston Diversity Index (HGDI) value for each MLVA-11 locus was calculated for the 600 *Brucella* isolates (see Table 1). Of the 600 *Brucella* isolates identified by the MLVA11 assay. *B. melitensis* was the majority with 398 strains (66.3%), followed by 97 (16.2%) of *B. abortus*, 58 (9.7%) of *B. suis*, and 47 (7.8%) of *B. canis*. Biovars 2 and 3 were dominant (99.0%, 394 strains) in *B. melitensis* with only four strains (1.0%) of biovar 1. Biovar 3 (75 strains) was the majority (77.3%) in *B. abortus*, with 17.5% of biovar 1 and five 5.2% of biovar 9. Biovar 1 and biovar 3 were almost half and half (48.3%) in *B. suis* with 28 strains each, and the remaining two strains (3.4%) were of biovar 2 (Additional file 2: Table S1).

**Table 1:** HGDI values of MLVA-11 loci for *Brucella* strains

Locus	Diversity Index (*n* = 600)	Confidence Interval	K	Max (pi)
Bruce19	0.867	0.843 – 0.892	16	0.200
Bruce12	0.758	0.713 – 0.802	10	0.333
Bruce18	0.703	0.646 – 0.760	7	0.457
Bruce06	0.62	0.548 – 0.692	6	0.552
Bruce11	0.567	0.465 – 0.669	8	0.638
Bruce08	0.533	0.441 – 0.624	5	0.648
Bruce21	0.513	0.424 – 0.603	6	0.657
Bruce43	0.512	0.424 – 0.600	4	0.657
Bruce42	0.503	0.406 – 0.601	6	0.676
Bruce55	0.395	0.290 – 0.501	4	0.762
Bruce45	0.364	0.271 – 0.458	4	0.771

Hunter Gaston Diversity Index (HGDI) was used to measure the variation of multiple locus variable-number tandem repeat locus, which ranges from 0.0 (no diversity) to 1.0 (complete diversity); Confidence Interval is Precision of the Diversity Index and expressed as 95% upper and lower boundaries; K: Number of different repeats present at this locus in this sample set; Max (pi): Fraction of samples that have the most frequent repeat number in this locus (ranging from 0.0 to 1.0); *n*: The number of the isolates according to the considered locus

**Fig. 1:**
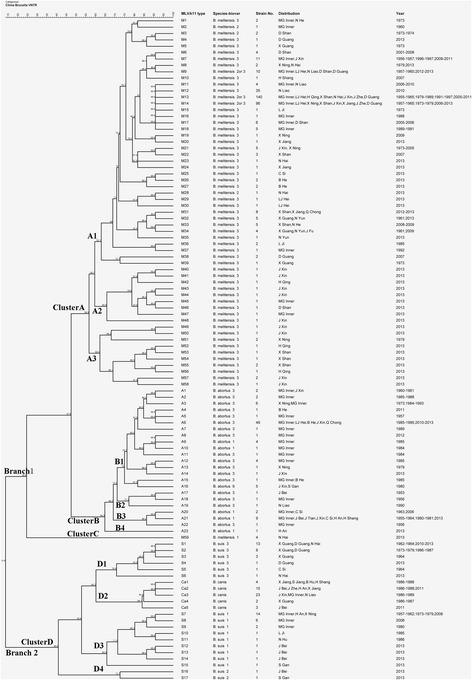
Cluster analysis by MLVA11 genotyping of the 600 *Brucella* strains from 1953 – 2013 in China. The cluster analysis was performed using the category coefficient and UPGMA (BioNumerics 5.1). B He:Hebei, B Hu:Hu Bei,C Si:Sichuan,D Guang:Guangdong,D Shan:Shandong, H An:Anhui,H Qing:Qinghai, H Shang:Shanghai, J Bei:Beijing, J Fu:Fujian, J Xin:Xinjiang,J Zhe:Zhejiang,L Ji:Jilin,LJ Hei:Heilongjiang, MG Inner:Inner Mongolia, N Hai:Hainan,N He:Henan, N He:Henan, N Hu:Hunan,N Liao:Liaoning, N Yun:Yunnan,S Gan:Gansu, Tianjin:J Tian, X Guang:Guangxi,X Jiang:Jiangxi, X Ning:Ningxia,X Shan:Shanxi

**Fig. 2:**
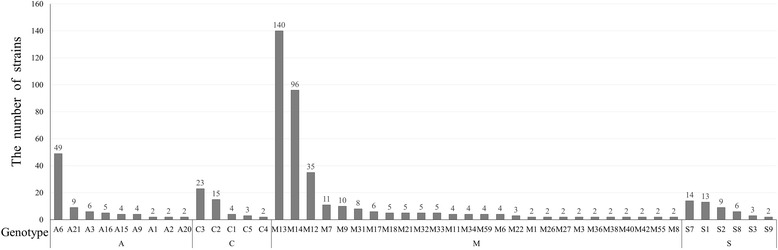
The relationship between the numbers of isolated strains and MLVA genotypes. The 58 singleton genotypes were not shown on the chart

The MLVA11 assay profiled the 600 *Brucella* isolates into 104 genotypes with 58 singleton genotypes (Fig. 1). The number of strains for each genotype was shown in Fig. 2. The largest group (59 genotypes) belonged to the 398 *B. melitensis* isolates, with 23, 17 and 5 genotypes representing *B. abortus*, *B. suis* and *B. canis* strains respectively (Fig. 1). These strains formed two branches (1 and 2) with 12 subclusters in total. Branch 1 was composed of *B. abortus* and *B. melitensis* whereas *B. suis* (biovars 1, 2 and 3) and *B. canis* belonged to branch 2. Cluster A, the largest with 394 *B. melitensis* biovars 2 and 3 isolates, were further divided into 4 subclusters (A1, A2, A3 and C) with a percentage of genetic similarity coefficient of 56.8%. Cluster B, including 97 *B. abortus* isolates, were further divided into four subclusters (B1, B2, B3 and B4) with a percentage of genetic similarity coefficient of 75.0%. Cluster C was small, containing only four strains of *B. melitensis* biovar 1. Branch 2 contained four clusters (D1, D2, D3 and D4) with a genetic similarity coefficient of 40.9%. The 58 *B. suis* isolates were scattered among clusters D1, D3 and D4. The 47 *B. canis* isolates together with the 28 *B. suis* biovar 3 isolates formed cluster D2 with a percentage of genetic similarity coefficient of 56.8% (Fig. 1).

Among the 104 genotypes were 12 main genotypes with more than two thirds 70.7% (424/600) of the 600 *Brucella* isolates. Specifically, 5 genotypes (M7, M9, M12, M13 and M14) accounted for 73.4% (292/398) of the *B. melitensis* strains. Two genotypes (A6 and A21) encompassed 59.8% (58/97) of *B. abortus*. Genotypes S1, S2, and S7 contained 62.1% (36/58) of the *B. suis* isolates whereas genotypes Ca2 and Ca3 covered 80.9% (38/47) of the *B. canis* strains (Fig. 2).

**Fig. 3:**
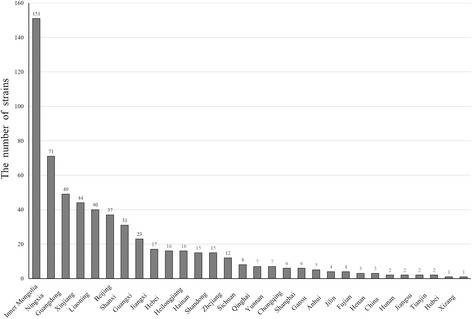
The relationship between numbers of isolated strains and isolated origins. The numbers marked in *solid lines* (isolated years) and *dotted lines* (isolated origins) represent the number of isolates that were isolated by their origins and year of isolation

The 600 strains could also be stratified by their geographical origin and time frame of isolation as shown in Fig. 3. All strains were from 4 regions: northern, northwestern, central and southern with details as follows. From the northern region, Inner Mongolia contributed the most nationwide (25.2%, 151/600), followed by Liaoning (6.7%, 40/600) and Heilongjiang (2.7%, 16/600) provinces. The numbers for the northwestern region were Xinjiang (7.3%, 44/600), Ningxia (11.8%, 71/600) and Shanxi (5.2%, 31/600). The central region were represented by Hebei (2.7%, 16/600), Beijing (6.2%, 37/600), Shandong (2.5%, 15/600), Jiangxi (2.8%, 17/600) and Zhejiang (2.0%, 12/600) provinces. Representing the southern region were Guangdong (8.2%, 49/600), Guangxi (3.8%, 23/600) and Hainan (2.5%, 15/600) provinces.

**Fig. 4:**
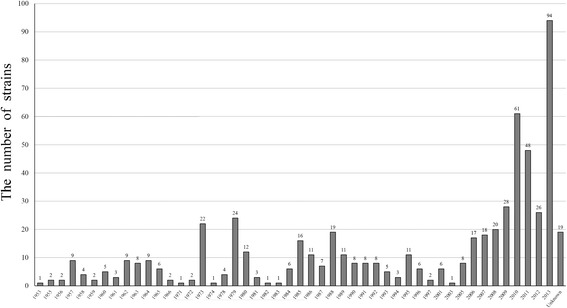
The relationship between the numbers of isolated strains and the isolated years

The time frame of strain isolation with more than 15 strains in a year was: 1973 (3.7%, 22/600), 1979 (4.0%, 24/600), 1985 (2.7%, 16/600), 1988 (3.2%, 19/600), 2006 (2.8%, 17/600), 2007 (3.0%, 18/600), 2008 (3.3%, 20/600), 2009 (4.7%, 28/600), 2010 (10.2%, 61/600), 2011 (8.0%, 48/600), 2012 (3.7%, 22/600) and 2013 (15.8%, 94/600), and unkown year (3.2%, 19/600) (Fig. 4).

The 600 *Brucella* strains were respectively isolated from blue sheep and pig (one strain each), camel (two), deer (three), yak (four), goat (12), swine (19), dog (47), sheep (81), cattle (90), human (272) and unknown (68).

### The time frame and geographic regions of the isolated strains

**Fig. 5:**
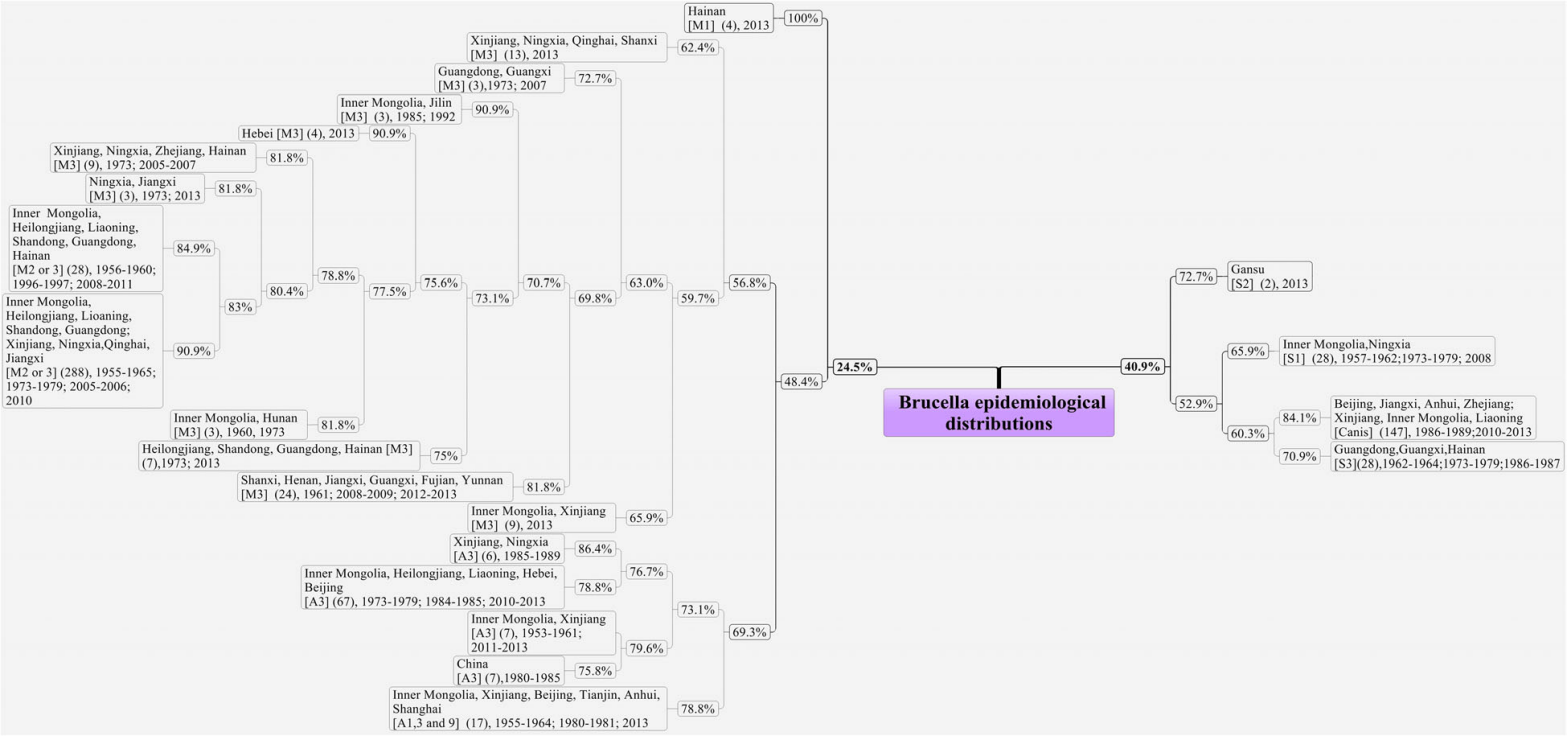
The epidemiological distributions of the 600 *Brucella* strains by MLVA11 genotyping. Note: “24.5%”, the percentage of genetic similarity coefficient was 24.5%; “Hainan [M1] (4), 2013”: “[M1]”, *Brucella melitensis* biotype 1; “(4)”, there was 4 strains with *Brucella melitensis* biotype 1; “2013”, the isolation year was 2013. “[M2 or 3]”, *Brucella melitensis* biotype 2 and 3; “[A1]”, *Brucella Abortus* biotype 1; “[A3]”, *Brucella Abortus* biotype 3; “[A9]”, *Brucella Abortus* biotype 9; “[S1]”, *Brucella suis* biotype 1; “[S2]”, *Brucella suis* biotype 2; “[S3]”, *Brucella suis* biotype 3; “[Canis]”, *Brucella canis*

A total of 398 *B. melitensis* isolates were divided into four clusters (A1, A2, A3, C) with a percentage of genetic similarity coefficient of 62.4%. Cluster A1 (372 strains belonging to *B. melitensis* biovars 2 and 3) were found in Inner Mongolia, Heilongjiang, Liaoning, Shandong, Guangdong, Xinjiang, Ningxia, Qinghai, Shanxi, Henan, Jiangxi, Guangxi, Fujian and Hainan provinces across years of 1955 – 1965, 1973 – 1979, 1991 – 1997, 2005 – 2008, and 2010 – 2013. Cluster A2 including 9 strains were isolated from Xinjiang, Inner Mongolia, Shandong and Qinghai provinces in 2013. Cluster A3 (13 isolates) were isolated from Xinjiang, Shanxi, Ningxia and Qinghai provinces in 2013 whereas cluster C (four isolates) was from Hainan province in 2013 (Fig. 5).

The four clusters (B1, B2, B3 and B4) consisted of 97 *B. abortus* isolates had a percentage of genetic similarity coefficient of 75.0%. The dominating cluster B1 included 81 *B. abortus* biovars 1 and 3 isolates from Inner Mongolia, Heilongjiang, Hebei, Xinjiang and Chongqing provinces during 1973 – 1979, 1984 – 1985 and 2010 – 2013. The three *B. abortus* biovar 3 isolates comprising cluster B2 were from Liaoning, Inner Mongolia and Beijing provinces during 1953 – 1956. By contrast of cluster B4 with just a single isolate from Anhui province in 2013, cluster B3 had 12 strains of *B. abortus* biovar 1 and 3 isolates from Xinjiang, Inner Mongolia, Tianjin, Sichuan, Anhui and Shanghai provinces during 1955 – 1964, 1980 – 1981 and 2013 (Fig. 5).

The D1, D3, D4 clusters (58 *B. suis* isolates) had a percentage of genetic similarity coefficient of 65.9%. Cluster D1 (28 biovar 3 *B. suis* strains) were isolated from Guangdong, Guangxi and Hainan provinces during 1962 – 1964, 1973 – 1979, 1986 – 1987 and 2010 – 2013. The second included 28 *B. suis* biovar 1 isolates, representing cluster D3, were from Inner Mongolia, Ningxia, Beijing, Jilin and Hunan provinces collected during 1957 – 1962, 1973 – 1979, 1986 – 1987, 2008 and 2013. D4 was a small cluster with only two biovar 2 isolates of *B. suis* from Gansu province in 2013 (Fig. 5).

With a percentage of genetic similarity coefficient of 84.1%, cluster D2 (47 *B. canis* isolates) was consisted of genotypes Ca2 and Ca3. Genotype Ca2 (31.9%, 15/47) were isolated from Zhejiang, Anhui and Jiangxi provinces during 1986 – 1988 and 2010 – 2011. Genotype Ca3 (48.9%, 23/47) were collected from Xinjiang, Inner Mongolia and Liaoning provinces during 1986 – 1989 (Fig. 5).

## Discussion

For many years, phenotypic characterization for *Brucella* strains was the only feasible way to provide epidemiological data with the disadvantage of lacking information related to their molecular background [8, 12]. Now with molecular tools, more options are available.

Although having higher discriminatory power than the MLVA11 assay, the MLVA16 assay with a set of 16 repeat loci is better for teasing out the difference of closely related isolates at fine-scale resolution whereas the MLVA11 assay is more suitable to analyze the phylogenetic and epidemiological relationships of *Brucella* strains because of its lesser degree of variation degree (HGDI values) [11, 13–18]. In accord with this notion, the genotyping results of MLVA11 assay have been consistent with those of MLVA71 assay genotyping [19], *Brucella*-specific IS711 element analysis [13], and whole-genome-based phylogeny studies [15, 16]. Therefore, we chose the MLVA11 assay to study the molecular epidemiology of *Brucella* isolates from China.

**Fig. 6:**
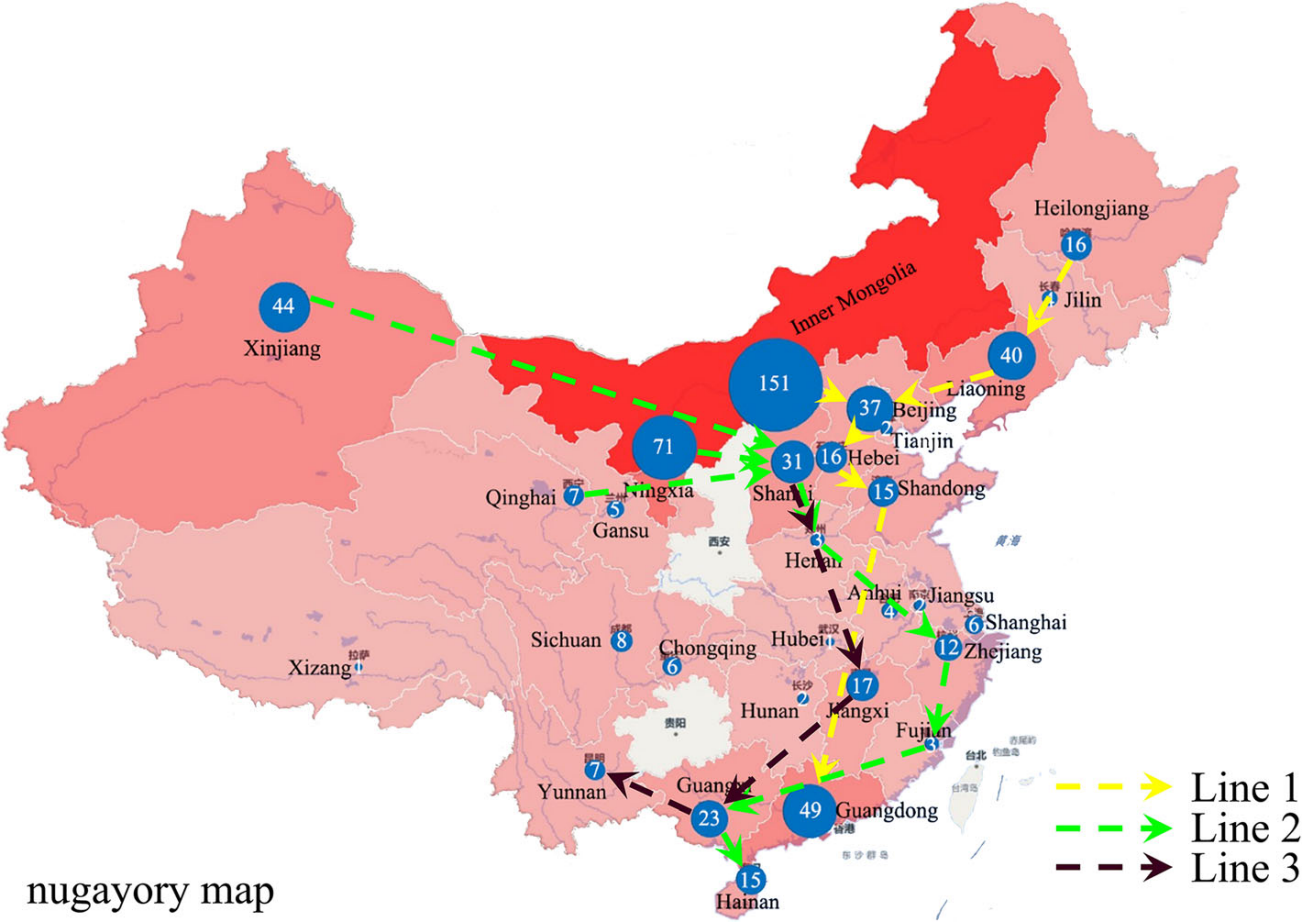
The sketch map of the 600 *Brucella* strains from different provinces in this study. Line 1 (*yellow line*): Inner Mongolia, Heilongjiang, Liaoning- Shandong- Hebei- Beijing-Guangdong provinces. Line 2 (*green line*): Xinjiang, Ningxia, Qinghai-Shanxi-Henan-Zhejiang-Guangxi-Fujian-Hainan provinces. Line 3 (*black line*) Shanxi-Henan-Jiangxi-Guangxi-Yunnan provinces. The size of the *blue circle* represented the total number of the isolated strains (*in a circle*). The sketch map does not represent an administrative map in the legal sense

The results of our cluster analysis using the MLVA11 assay were meaningful in terms of the isolation time frame, host and region of these strains. For the past 60 years the dominant *Brucella* species and biovars were *B. abortus* biovars 1 and 3, *B. melitensis* biovars 2 and 3, *B. suis* biovar 1 and 3, and *B. canis*. The cluster analysis results of the 600 *Brucella* isolates correlated well with their genetic variation patterns and the Brucellosis epidemics. For instances, before 1980 most *B. abortus* biovars 1 and 3 isolates were seen in the northern region such as Inner Mongolia, Xinjiang, Gansu and Ningxia provinces. However, after 2000 *B. abortus* strains have emerged in the central and southern regions like Hebei, Beijing, Chongqing and Zhejiang provinces. According to results of our MLVA analysis, there were eight epidemic periods across years of 1955 – 1959, 1962 – 1969, 1971 – 1975, 1977 – 1983, 1985 – 1989, 1992 – 1997, 2000 – 2008 and 2010 – 2013 (Figs. 1, 5 and 6) with each peak at years of 1958, 1966, 1973, 1980, 1987, 1996, 2007 and 2013. Most of the involved livestock were either raised or processed in the northern and northwestern regions in China. Results of the MLVA analysis (Figs. 1, 5 and 6) suggested that there were three predominant but different lines of *Brucella* transmission in China. The first line (Line 1) involved Inner Mongolia, Heilongjiang, Liaoning, Shandong, Hebei, Beijing and Guangdong provinces. The second line included places in Xinjiang, Ningxia, Qinghai, Shanxi, Henan, Zhejiang, Guangxi, Fujian and Hainan provinces (Line 2). And the third line was represented by Shanxi, Henan, Jiangxi, Guangxi and Yunnan provinces (Line 3) (Fig. 6). As to the species and biovar, most *B. suis* biovar 1 isolates were from the northern region including Inner Mongolia and Ningxia provinces while the majority of the *B. suis* biovar 3 isolates were from the southern region, such as Guangdong and Guangxi provinces.

Considering the finding that most of the cluster A1 strains (93.5% of the *B. melitensis* biovars 2 and 3, with a percentage of genetic similarity coefficient of 62.4%) have been existing in different provinces for a long time (from 1953 to 2013) whereas clusters A2 and A3 isolates started to appear in 2013 in Inner Mongolia, Xinjiang and Shanxi provinces (Fig. 1), questions arise such as whether these cluster A2 and A3 strains are variants of other biovars, or have they been causing diseases all these years but without being isolated, what is its impact on trend of Brucellosis in China, and so on. All such questions remain to be answered in the future.

## Conclusions

Taken together, our study showed the MLVA is a reliable monitoring method for the molecular epidemiology of Brucellosis relating to strain phylogeny and the regional and time distribution of the disease, potentially valuable for predicting the epidemic trend of Brucellosis in China.

## Additional files

Additional file 1: Multilingual abstract in the five official working languages of the United Nations. (PDF 972 kb)

Additional file 2: Table S1. MLVA-11 genotyping and the data of 600 *Brucella* strains in China. (XLS 166 kb)
