# Supplementary material for: Multi-locus variable-number tandem repeat analysis of Chinese Brucella strains isolated from 1953 to 2013
Source: Infect Dis Poverty. 2017 May 2;6:89. doi: 10.1186/s40249-017-0296-0 (PMC5412030; doi:10.1186/s40249-017-0296-0)
Supplement: Supplementary file 1 — Multilingual abstract in the five official working languages of the United Nations. (PDF 972 kb) [file 40249_2017_296_MOESM1_ESM.pdf]

Translation of the abstract into the five official working languages of the United Nations

تحليل متكرر ومتغير العدد ومتعدد الأماكن عن سلالات البروسيلا الصينية المُستقى من 1953 إلى 2013

قوه تشونغ تيان، بو يون تسوى، دونغ-ري بياو، هونغ يان تشاو، لان يو لى، شى ليو، بى شياو، تشونغ تشى تشاو، لى تشينغ شو، هاي جيانغ وتشن جون لي

ملخص

خلفية: كان البروسيلا مرضا يصيب الإنسان والمواشي بسبب سلالات البروسيلا، ومسببات الأمراض ذات الأولوية من الفئة "ب" طبقا لتصنيف المركز الأمريكي لمكافحة الأمراض (CDC). بعد أن حُدد المرض على أنه مرض ذو أولوية يصيب البشر والماشية، فإن تزايد الإصابة به في السنوات الأخيرة في الصين يحتاج إلى تدابير الرقابة العاجلة لمكافحة هذا المرض ولكن الخلفية الجزيئية الهامة لرصد وبائيات سلالات البروسيلا على المستوى الوطني لا تزال غير موجودة.

الطرق: تم جينيا تنميط ما مجموعه 600 مستفردة من البروسيلا من المستفردات التي تم جمعها خلال 60 عاما (من 1953 إلى 2013) في الصين بواسطة تحليل متكرر ومتغير العدد ومتعدد الأماكن (MLVA)، وتم حساب درجة تغير أماكن MLVA11 بواسطة قيم مؤشر التنوع لغاستون هنتر (HGDI). جُهزت المخططات والخريطة باستخدام إكسل 2013، وأجري التحليل العنقودي والتوزيع الوبائي باستخدام BioNumerics (الإصدار 5.1).

النتائج: أُدرجت المستفردات الـ 600 الممثلة للبروسيلا في 104 نمط جيني مع 58 من النمط الجيني المفرد بواسطة فحص MLVA11، بما في ذلك النوع الحيوي من البروسيلا المالطية 2 و 3 (الأنماط الجينية الخمسة الرئيسية)، والنوع الحيوي من البروسيلا المجهضة و 3 (اثنان من الأنماط الجينية الرئيسية)، والنوع الحيوي من بروسيلا الخنازير 1 و3 (الأنماط الجينية الثلاثة الرئيسية)، البروسيلا الكلبية (اثنان من الأنماط الجينية الرئيسية) على التوالي. في حين وُجد معظم النوع الحيوي من بروسيلا. الخنازير 1 والنوع الحيوي 3 على التوالي في المحافظات الشمالية والمحافظات الجنوبية، فإن سلالات البروسيلا المالطية والبروسيلا المجهضة كانت هي المنتشرة في الصين. تم العثور على البروسيلا الكلبية فقط في الحيوانات دون الابلاغ عن أي إصابات بشرية. ظهرت ثمانية أوبئة قوية للبروسيلا خلال 60 سنة بين عام 1955-1959 :1953-2013، 1969-1962، 1971-1975، 1977-1983، 1985-1989، 1992-1997، 2000-2008 و2010-2013 في الصين.

الخاتمة: تمتلك الحمى المالطية أنماطا فريدة وبائية جزيئية مع التوزيع المكاني والزماني المحددة وفقا لـ MLVA.

Translated from English version into Arabic by Mansour Awwad, through

TRANSLATORS
WITHOUT BORDERS

1953-2013 年中国分离的布鲁氏菌多位点串联重复序列基因分析

摘要

引言： 布鲁氏菌病是由布鲁氏菌引起的人畜共患性传染病，美国 CDC 定为 B 类病原体。近几年，中国的布病发病率呈现明显的上升趋势，成为威胁着人类和家畜主要传染病，迫切需要采取控制措施，但是目前缺乏全国布鲁氏菌菌株的分子流行病学监测背景。

方法： 我们应用多位点串联重复序列基因分型方法分析中国 60 年来（1953—2013）收集的 600 株代表性布鲁氏菌的基因特征，采用 Hunter Gaston Diversity Index (HGDI)指数对 11 个位点参数变异度进行分析，图表和地图采用 Excel 2013 进行加工处理。应用 BioNumerics (version 5.1)软件进行聚类分析和分子流行病学分布规律研究，

结果：600 株代表性布鲁氏菌菌株分为 104 个 MLVA 基因型，包括 58 个单个基因型。主要流行菌株包括布鲁氏菌羊种 2 和 3 型菌株（主要是 5 个基因型）；牛种 1 和 3 生物型菌株（主要包括 2 个基因型）；猪种 1 和 3 生物型（主要包括 3 个基因型）；犬种菌（主要是 2 个基因型）。多数猪种生物型 1 和 3 型菌株分布在中国的北部和南部地区，羊种菌和牛种菌是中国主要感染菌。犬种菌主要从动物组织分离到，人类没有病例报告。中国布病 60 年来（1953–2013）年出现过 8 次流行，分别是 1955–1959, 1962–1969, 1971–1975, 1977–1983, 1985–1989, 1992–1997, 2000–2008 和 2010–2013。

结论：依据布鲁氏菌多位点串联重复序列基因特征，中国的布病疫情具有独特的地域和时间分子流行病学特征。

Translated from English version into Chinese by Guo-Zhong Tian

Analyse multilocus du polymorphisme de séquences répétées en tandem des souches de *Brucella* en Chine isolées entre 1953 et 2013

Guo-Zhong Tian, Bu-Yun Cui, Dong-Ri Piao, Hong-Yan Zhao, Lan-Yu Li, Xi Liu, Pei Xiao, Zhong-Zhi Zhao, Li-Qing Xu, Hai Jiang et Zhen-Jun Li

Résumé

Contexte : La brucellose était une maladie commune touchant les humains ainsi que le bétail et qui est causée par les souches *Brucella*, les pathogènes prioritaires de catégorie B selon le centre de lutte contre les maladies des É.-U. [Center for Disease Control (CDC)]. Identifiée comme une maladie prioritaire chez les humains et le bétail, la hausse de son incidence au courant des dernières années en Chine nécessite des mesures de contrôle urgentes relatives à cette maladie, mais la base moléculaire importante pour surveiller l'épidémiologie des souches *Brucella* au niveau national est toujours lacunaire.

Méthodes : Un total de 600 isolats de *Brucella* prélevés sur 60 ans (entre 1953 et 2013) en Chine ont fait l'objet de génotypage au moyen d'analyse multilocus du polymorphisme des séquences répétées en tandem (MLVA), et le degré de variation de loci MLVA11 a été calculé selon les valeurs de l'indice de diversité Hunter Gaston (HGDI). Les graphiques et les cartes ont été traités au moyen d'Excel 2013, et l'analyse des groupes désignés ainsi que la distribution épidémiologique ont été réalisées au moyen de BioNumerics (version 5.1).

Résultats : Les 600 isolats représentatifs de Brucella ont présenté 104 génotypes, avec 58 génotypes

uniques selon l'analyse MLVA11, y compris les biovars 2 et 3 *B. melitensis* (cinq principaux génotypes), les biovars 1 et 3 *B. abortus* (deux principaux génotypes), les biovars 1 et 3 *B. suis* (trois principaux génotypes) et la souche *B. canis* (deux principaux génotypes), respectivement. Alors que la plupart des biovars 1 et 3 *B. suis* ont été trouvés dans les provinces du Nord et du Sud respectivement, les souches *B. melitensis* et *B. abortus* étaient dominants en Chine. La brucellose canine a été retrouvée seulement dans les animaux, et aucun cas n'a été rapporté chez les humains. Huit pics épidémiques de brucellose ont été constatés au courant des 60 années entre 1953 et 2013 : 1955-1959, 1962-1969, 1971–1975, 1977–1983, 1985–1989, 1992–1997, 2000–2008 et 2010–2013 en Chine.

Conclusions : Selon l'analyse MLVA, la brucellose présente des tendances épidémiologiques moléculaires qui lui sont propres, avec une distribution spatiale et temporelle spécifique.

Translated from English version into French by Edith Emilie Mercier, through

TRANSLATORS
WITHOUT BORDERS

Мильтилокусный анализ VNTR-последовательностей китайских штаммов *Brucella,* выделенных с 1953 г. по 2013 г.

Го-Чжун Тянь (Guo-Zhong Tian), Бу-Юнь Цуй (Bu-Yun Cui), Дун-Жи Пяо (Dong-Ri Piao), Хун-Янь Цзао (Hong-Yan Zhao), Лань-Юй Ли (Lan-Yu Li), Си Лю (Xi Liu), Пэй Сяо (Pei Xiao), Чжун-Чжи Чжао (Zhong-Zhi Zhao), Ли-Цин Сюй (Li-Qing Xu), Хай Цзян (Hai Jiang) и Чжэнь-Цзюнь Ли (Zhen-Jun Li)

Аннотация

Краткое описание. Бруцеллёз был распространённым заболеванием человека и домашнего скота, возбудителем которого являются штаммы *Brucella* – патогены категории B в соответствии с классификацией Центра контроля заболеваемости США (CDC). Бруцеллёз был признан болезнью первостепенной важности для людей и животных. Растущее число случаев заболеваний за последние годы в Китае требует принятия срочных мер, однако молекулярных данных, необходимых для наблюдения за эпидемиологией штаммов *Brucella* на национальном уровне, по-прежнему недостаточно.

Методы. Собранные в Китае за 60 лет (с 1953 г. по 2013 г.), 600 изолятов *Brucella* были генотипированы с использованием мультилокусного анализа VNTR-последовательностей (MLVA). Степень вариабельности локусов MLVA11 была определена по величинам индекса Хантера-Гастона (HGDI). Диаграммы и карта были обработаны с помощью Excel 2013. Кластерный анализ и эпидемиологическое распределение были выполнены с использованием BioNumerics (версия 5.1).

Результаты. Согласно анализу MLVA11, 600 репрезентивных изолятов *Brucella* относятся к 104 генотипам с 58 одинарными генотипами, включая биовары 2 и 3 *B. melitensis* (пять основных генотипов), биовары 1 и 3 *B. abortus* (два основных генотипа), биовары 1 и 3 *B. suis*

(три основных генотипа) и *B. canis* (два основных генотипа) соответственно. В то время как большинство биоваров 1 и 3 *B. suis* были найдены соответственно в северных и южных провинциях, в Китае преобладали штаммы *B. melitensis* и *B. abortus*. Бруцеллёз собак был обнаружен только у животных, случаев заболеваний людей не было. За 60 лет с 1953 года по 2013 год в Китае было восемь пиков эпидемий бруцеллёза: 1955–1959 гг., 1962–1969 гг., 1971–1975 гг., 1977–1983 гг., 1985–1989 гг., 1992–1997 гг., 2000–2008 гг. и 2010–2013 гг.
Выводы. Согласно анализу MLVA, бруцеллёз обладает собственными уникальными эпидемиологическими характеристиками со специфическим распределением в пространстве и времени.

Translated from English version into Russian by Natalia Potashnik, through

TRANSLATORS
WITHOUT BORDERS

Análisis de repeticiones en tándem de números variables en múltiples loci en cepas de *Brucella* aisladas en China entre 1953 y 2013

Guo-Zhong Tian, Bu-Yun Cui, Dong-Ri Piao, Hong-Yan Zhao, Lan-Yu Li, Xi Liu, Pei Xiao, Zhong-Zhi Zhao, Li-Qing Xu, Hai Jiang y Zhen-Jun Li

Resumen

Antecedentes: La brucelosis era una enfermedad común de los humanos y el ganado, producida por cepas de *Brucella*, clasificados como patógenos de prioridad categoría B por el Centro para la Prevención y Control de Enfermedades de los Estados Unidos (CDC). Identificada como una enfermedad prioritaria del ser humano y el ganado, la creciente incidencia en China en los últimos años requiere de medidas de control urgentes para esta enfermedad, pero todavía no existe a nivel nacional el entorno molecular tan importante para el monitoreo de la epidemiología de cepas de *Brucella*.
Métodos: Se realizó genotipificación por análisis de repeticiones en tándem de números variables en múltiples loci (MLVA) a un total de 600 cepas aisladas de *Brucella* recolectadas en el transcurso de 60 años (de 1953 a 2013) en China y se calculó el coeficiente de variación del loci MLVA11 utilizando los valores del índice discriminatorio de Hunter-Gaston (HGDI). Los gráficos y mapas se procesaron con Excel 2013 y el análisis de grupos y distribución epidemiológica se realizó con BioNumerics (versión 5.1).
Resultados: Con el ensayo de MLVA11 los 600 aislados representativos de *Brucella* cayeron dentro de 104 genotipos con 58 genotipos únicos, incluido *B. melitensis* biovares 2 y 3 (cinco genotipos principales), *B. abortus* biovares 1 y 3 (dos genotipos principales), *B. suis* biovares 1 y 3 (tres genotipos principales) y *B. canis* (dos genotipos principales) respectivamente. Si bien la mayoría de *B. suis* biovar 1 y biovar 3 se encontraron en provincias del norte y del sur respectivamente, las cepas de *B. melitensis* y *B. abortus* fueron dominantes en China. La brucelosis canina solo se

encontró en animales sin casos reportados en seres humanos. Emergieron ocho picos de epidemias de brucelosis en el transcurso de los 60 años entre 1953 y 2013 en China: 1955–1959, 1962–1969, 1971–1975, 1977–1983, 1985–1989, 1992–1997, 2000–2008 y 2010–2013.
Conclusiones: De acuerdo con el MLVA, la brucelosis tiene patrones epidemiológicos moleculares únicos con distribución espacial y temporal específicas.

Translated from English version into Spanish by Maria Alejandra Aguada, through

TRANSLATORS
WITHOUT BORDERS
